# Effect of combining photoinitiators on cure efficiency of dental resin-based composites

**DOI:** 10.1590/1678-7757-2020-0467

**Published:** 2021-07-23

**Authors:** Lucas Lara, Mateus Garcia Rocha, Livia Rodrigues de Menezes, Américo Bortolazzo Correr, Mario Alexandre Coelho Sinhoreti, Dayane Oliveira

**Affiliations:** 1 Universidade Estadual de Campinas – UNICAMP Faculdade de Odontologia de Piracicaba Departamento de Odontologia Restauradora PiracicabaSP Brasil Universidade Estadual de Campinas – UNICAMP, Faculdade de Odontologia de Piracicaba, Departamento de Odontologia Restauradora, Piracicaba, SP, Brasil.; 2 University of Florida Division of Operative Dentistry Department of Restorative Dental Sciences GainesvilleFL United States University of Florida, Division of Operative Dentistry, Department of Restorative Dental Sciences, Gainesville, FL, United States.; 3 Universidade Federal do Rio de Janeiro Instituto de Macromoléculas Professora Eloisa Mano Rio de JaneiroRJ Brasil Universidade Federal do Rio de Janeiro, Instituto de Macromoléculas Professora Eloisa Mano, Rio de Janeiro, RJ, Brasil.

**Keywords:** Dental photoinitiators, Polymerization, Raman spectroscopy, Spectrophotometry

## Abstract

Camphorquinone is the most conventionally used photoinitiator in Dentistry. Although different alternative photoinitiators have been proposed, no photoinitiator was capable of completely substituting camphorquinone. The combination of photoinitiators has been considered the best alternative.

**Objectives:**

To evaluate the effect of combining Norrish type I and II photoinitiators on the cure efficiency of dental resin-based composites.

**Methodology:**

Experimental composites were produced containing different photoinitiator systems: Norrish type I-only, mono-alkyl phosphine oxide (TPO); Norrish type II-only, camphorquinone (CQ); or its combination, CQ and TPO, in a 1: 1 molar ratio. UV-vis absorption spectrophotometry was performed to assess the consumption of each photoinitiator after curing (n=3). A multi-wave LED (Bluephase^®^ G2, Ivoclar Vivadent) was pre-characterized and used with a radiant exposure of 24 J/cm2. The degree of conversion was evaluated by Raman spectrometry, and the elution of the monomers by nuclear magnetic resonance analysis (n=3). Data were analyzed using ANOVA and Tukey's test (α=0.05; β=0.2).

**Results:**

The combination of CQ and TPO increased the consumption of the photoinitiator system compared to CQ-only (p=0.001), but presented similar consumption compared to TPO-only (p=0.52). There was no significant difference in the degree of conversion between the composites regardless of the photoinitiator system (p=0.81). However, the elution of the monomers was reduced when both photoinitiators were combined. TPO-based material presented the highest elution of monomers.

**Conclusions:**

The combination of the photoinitiator systems seems to be beneficial for the cure efficiency of dental resin-based composites.

## Introduction

Light cured resin-based materials are composed of monomers that, after exposure to light, form a polymer. This process of building a polymer through the combination of monomers is called polymerization. When polymerization is triggered by a physical medium, such as light, this process is called photopolymerization.^1^ Photoactivation promotes the excitation of the photoinitiators. After being excited, the photoinitiators react, generating free radicals.^2^ The free radicals, in turn, are responsible for breaking the double bonds of the monomers. So that for the chemical stabilization of the molecule, the monomers bind together, forming larger units and the polymers.^1^

Camphorquinone is the most used photoinitiator system in the manufacture of dental resin-based materials since 1970.^3^ Camphorquinone is a Norrish type II photoinitiator. This classification is due to the need to be combined with a reducing agent to generate free radicals and initiate the polymerization reaction.^4^^-^^6^ In the case of camphorquinone, the most common reducing agents are tertiary amines.^2^

On the other hand, Norrish type I photoinitiators are capable of generating free radicals after photoactivation without the need for a reduction agent. Generation of free radicals occurs through the self-cleavage of the photoinitiator molecule itself, creating at least two free radicals from this self-cleavage. The mono-alkyl phosphine oxide (TPO) is a well-known tested Norrish type I photoinitiator in Dentistry.^5^^-^^6^

Several studies have demonstrated the curing efficiency of mono-alkyl phosphine oxide for application in some dental resin materials.^3^^-^^7^ However, it is also known that there are limitations for its use combined with other photoinitiators, such as camphorquinone. Mono-alkyl phosphine oxide is a much more reactive molecule than camphorquinone. The mono-alkyl phosphine oxide can generate two active free radicals that can initiate the polymerization reaction. At the same time, the camphorquinone-based system, combined with a reducing agent, is only capable of producing one active free radical.^5^^-^^8^ On the other hand, camphorquinone is activated by the blue wavelength spectrum, while the mono-alkyl phosphine oxide, by the violet wavelength spectrum.^9^ The blue wavelength spectrum can penetrate deeper through the composite compared to the violet wavelength spectrum. Thus, for resin-based materials that need to be photoactivated to a certain depth or thickness, mono-alkyl phosphine oxide may present a certain disadvantage compared to the camphorquinone-based system.^4^^,^^7^^,^^9^ Still, the quality of the polymer not only depends on the degree of conversion the material can achieve, but the kinetics of conversion from the photoinitiators or their combination. Thus, the monomer elution is an important parameter to evaluate the quality of the polymeric chain formed with the presence – or not – of branches or reticulations between the polymers.^10^^,^^11^

Recent studies have shown the combination of Norrish type I and II photoinitiators can be even more efficient compared to Norrish type I photoinitiators.^7^^,^^9^ This fact seems to be related to a possible synergy effect when the two photoinitiator systems are combined.^9^ However, further research on the impact of combining Norrish type I and II photoinitiator have not yet been conducted. Thus, this study aimed to evaluate the effect of combining Norrish type I and II photoinitiators on the cure efficiency of dental resin-based composites. The tested hypotheses were: (1) The combination of Norrish type I and II photoinitiators increases the consumption of the photoinitiator system; (2) The combination of Norrish type I and II photoinitiators increases the degree of conversion of dental resin-based composites; and (3) the combination of Norrish type I and II photoinitiators produces less elution of the monomers.

## Methodology

### Experimental resin-based composites

**Figure 1:**
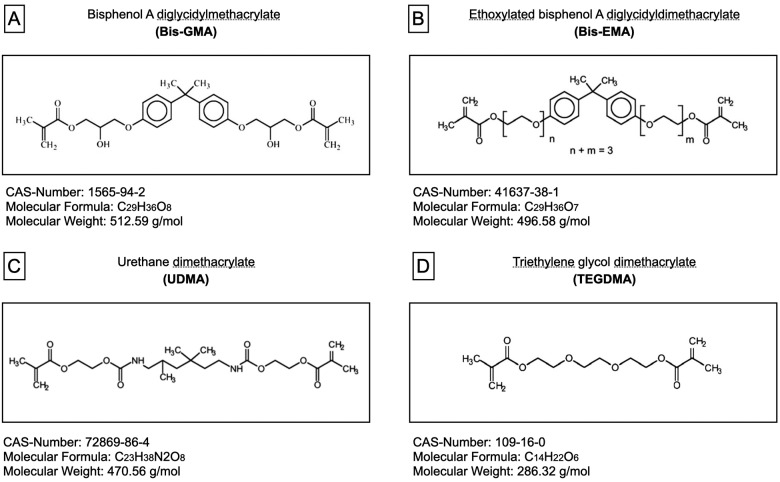
Chemical information of the monomers: (A) Bis-GMA; (B) Bis-EMA; (C) UDMA; (D) TEGDMA

Table 1 lists the monomers and filler particles and their concentrations used in the experimental dental composites. Figure 1 also illustrates the chemical details of each monomer used in the composition. The monomers were blended using a centrifugal mixing device (SpeedMixer, DAC 150.1 FVZ- K, Hauschild Engineering, Hamm, North Rhine-Westphalia, Germany). To this resin blend, different molar concentrations of CQ-amine (1:1)^12^ and TPO were added as described in Table 2.^7^ Subsequently, the filler particles were added, first by pre-mixing the fumed silica filler with the monomer blend for 30 seconds at 3,000 rpm, followed by the barium borosilicate glass filler for 1 minute at 3,500 rpm. Then, each resin-based composite was mixed one final time for 1 minute at 3,500 rpm under vacuum to eliminate porosities.

**Table 1:** Experimental composites composition

Material	Chemical*	wt%	Manufacturer
Monomers	Bis-GMA	25	Esstech Inc, Essington, PA, USA
	Bis-EMA	34,5	
	UDMA	34,5	
	TEGDMA	6	
Fillers	0.05 µm Silica	13	Nippon Aerosil Co Ltd, Tokyo, Japan
	0.7µm BaBSiO^2^	52	Esstech Inc, Essington, PA, USA

* Bisphenol A glycidyl methacrylate (BisGMA), Ethoxylated bis-phenol A methacrylate (BisEMA), Urethane dimethacrylate (UDMA), Triethylene glycol dimethacrylate (TEGDMA).

**Table 2:** Photoinitiator systems evaluated

Photoinitiator	Molar Ratio	wt %		
System	CQ:TPO	CQ	EDMAB	TPO
CQ	1:0	0.2	0.2	0
TPO	0:1	0	0	0.4
CQ:TPO	1:1	0.1	0.1	0.2

Molecular weight: CQ = 166.22 g/Mol; EBMAB = 193.98 g/Mol; TPO = 348,37 g/Mol.

### Curing light characterization

A multi-wave curing light (Bluephase G2, Ivoclar Vivadent, Schaan, Liechtenstein) with a standardized tip (9 mm diameter) was used in this study. First, the light tip active area of emission was measured using a bean profile.^7^^,^^9^ The output power (mW) was measured with a calibrated power meter (Ophir Optronics, Har-Hotzvim, Jerusalem, Israel). The light irradiance (mW/cm^2^) was calculated by dividing the output power by the area of the light tip. The spectral distribution was obtained by using a pre-calibrated spectrometer (USB2000, Ocean Optics, Dunedin, FL, USA), and the spectral distribution data were integrated using Origin 6.0 software (OriginLab, Northampton, MA, USA).

The Bluephase^®^ G2 had an active area of emission of 0.646 cm^2^. The mean irradiance of the Bluephase^®^ G2 was 1195 mW/cm^2^ ± 17 mW/cm^2^ and had a total radiant exposure of 24 J/cm^2^ ± 0.5 J/cm^2^ after 20 seconds of exposure, with 19.4 J/cm^2^ ± 0.6 J/cm^2^ being generated over the blue wavelength range of 420-495 nm and 4.6 J/cm^2^ ± 0.3 J/cm^2^ over the violet wavelength range of 380-420 nm. The specimens had a surface area of 0.196 cm^2^. The mean irradiance received by the specimens was 888 mW/cm^2^ ± 10 mW/cm^2^ and had a total radiant exposure of 18 J/cm^2^ ± 0.2 J/cm^2^ after 20 seconds of exposure, with 15 J/cm^2^ ± 0.1 J/cm^2^ being generated over the blue wavelength range of 420-495 nm and 3 J/cm^2^ ± 0.0 J/cm^2^ over the violet wavelength range of 380-420 nm.

**Figure 2:**
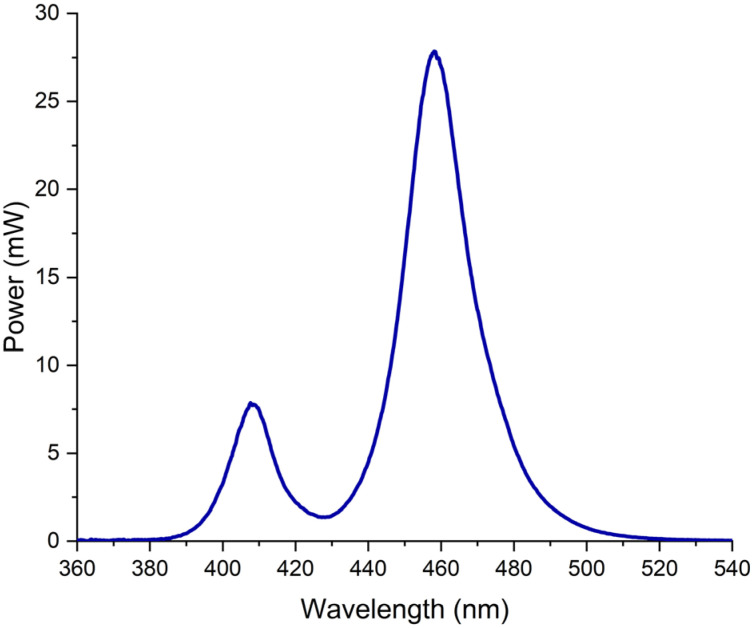
Absolute radiant emittance (mW/cm2) x wavelength emittance (nm) for the multi-wave LED

Figure 2 illustrates the spectral power (mW) distribution according to each wavelength (nm). As it can be observed, the Bluephase^®^ G2 is a dual peak multi-wave curing light, with one LED chip emitting “violet” light with peak at 410 nm, and three LED chips emitting “blue light” with peak at 460 nm. The reason for using a multiwave curing light in this study is because most of the absorption of CQ is within the 430-490 nm range, or the “blue light” range, with absorption peak approximately at 470 nm, whereas the absorption peak of TPO is mainly in the near UV-A region and extends to the violet spectrum range (380-420 nm).

### Photoinitiators consumption by absorption spectrophotometric analysis

First, a calibration curve was created by first preparing a set of standard solutions with known concentrations of each photoinitiator and its combination. All solutions were prepared with 0.1 ml of the monomer blend presented in Table 1 as the diluent. For each solution, the absorbance at a similar wavelength was measured, and a graph of absorbance against concentration was plotted. All spectra were collected in the 200-600 nm wavelength range using a UV–Vis spectrophotometer (U-2450, Hitachi High- Technologies, Chiyoda, Tokyo, Japan). The spectra were collected using a disposable cell with a path length of 1 cm. Then, an initial spectrophotometric analysis of each photoinitiator diluted in 0.1 ml of the same monomer blend at the concentrations stated in Table 2 was performed to confirm the initial concentration of the photoinitiators tested and the accuracy of the calibration curve. The amount of 0.1 ml was chosen as it was the exact same amount of monomer blend used to produce the samples used in the other analyses in this study. Right after the initial spectrophotometric analysis, a secondary analysis was performed immediately after polymerization to evaluate theconsumption of the photoinitiators. Thus, before collecting the second spectra, the resin-based material inside the disposable cell was light-cured with 24 J/cm2 of radiant exposure. Then, the spectra were collected within the same parameters as previously described (n=3). The final concentration of each photoinitiator was verified using the concentration curve. The consumption in percentage was calculated for each solution containing the different photoinitiators or their combinations.

### Degree of conversion analysis

The cure efficiency for each resin was measured using a µ-Raman spectrometer (Xplora, Horiba, Kyoto, Japan) (n=3). Each experimental resin-based composite was placed in a silicon rubber mold (Ø=5 mm, 1 mm thick) sandwiched between two polyester strips. First, the unpolymerized blends were scanned, then light cured with 24 J/cm^2^, and immediately rescanned. All light curing procedures were performed with the curing light tip positioned in the center of the specimen. All spectra were obtained by the coaddition of 32 scans at a resolution of 4 cm^-1^. Data were exported to a software (SpectraGryph 1.2, Effemm, Oberstdorf, Germany), and the derivative of the 1,610 cm-1 and 1,640 cm-1 peaks corresponded to the phenyl CC peak and the vinyl CC peak, respectively. The degree of conversion (DC) was calculated using the equation:

C=100×[1−(pRolimerizationuRnpolymeized)]

where “R” is the peak absorption area ratio at 1640 cm^−1^/1610 cm^−1^.

### Monomer elution

1H NMR experiments were carried out using a Varian Mercury (Palo Alto, CA, USA), operating at 300 MHz. To obtain the spectra of each reference monomer, 0.01 g of the monomer were dissolved in 0.7 mL of deuterated chloroform. The spectra were analyzed using the MestreLab Nova software, and the molecular structure elucidation was carried out according to the signals obtained in each spectrum.

All samples from the degree of conversion analysis were weighted and immediately immersed in 1 ml of dichloromethane (Sigma Aldrich, St. Louis, MO, United States) in sealed glass vials for 14 days. Then, the solvent was evaporated, and the monomer elution dissolved in 1 ml of deuterated chloroform (Sigma Aldrich, St. Louis, MO, United States). All content was then transferred to an NMR tube and analyzed by nuclear magnetic resonance.

The signals of each sample were overlapped and based on the integration of the peaks of 1H, the concentration of each monomer on the solution was determined. First, aliphatic monomers were separated from aromatic monomers where (A) = peaks at 7.15 / 6.85 ppm correspond to CH in the aromatic rings (four 1H per molecule) and (B) = peaks at 6.15 / 5.60 ppm correspond to CH2 in methacrylate functional groups (two 1H per molecule). If (A) is present, assume (A)/(B) ratio of 2:1. The exceeding area for (B) corresponds to methacrylate in aliphatic molecules. Second, Bis-GMA monomer was separated from Bis-EMA monomer where (C) = peak at 4.50 ppm only exists in Bis-EMA (CH2 on short arm – two 1H per molecule) and (D) peak at 2.73 ppm only exits in Bis-GMA (OH on backbone – two 1H per molecule). If 2.73 peak is present, assume B/D ratio of 1:1. The exceeding are for B (only the aromatic portion) corresponds to methacrylate in Bis-GMA. Third, TEGDMA monomer was separated from UDMA monomer, where (E) = peaks at 0.92 ppm (CH3 in butyl – six 1H per molecule) and 0.88 ppm (CH3 in propyl – three 1H per molecule). If (E) is present, assume E/B ratio of 9:2. The exceeding area for B (only the aliphatic portion) corresponds to methacrylate in TEGDMA.

### Statistical analyses

Power analysis was conducted to determine the sample size for each experiment to provide a power of at least 0.8 at a significance level of 0.05 (β=0.2). Data were checked for normality by Shapiro-Wilk's test and homoscedasticity of variances by Levene's test. All data were analyzed using a one-way ANOVA test, followed by Tukey's post-hoc test for multiple comparisons. A 95% significance level was considered for all analyses.

## Results

### Photoinitiators consumption

**Figure 3:**
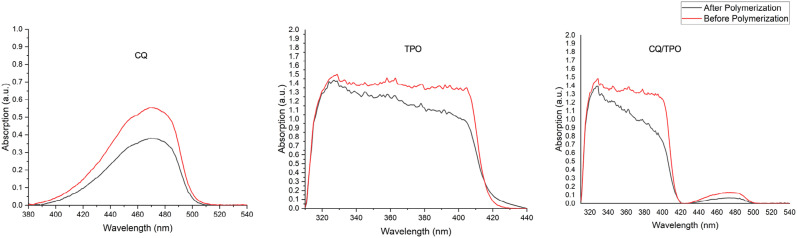
Absorbance (L mol – 1 cm – 1) x wavelength (nm) for each solution before and after polymerization

Figure 3 illustrates the absorbance of each photoinitiator plotted against the wavelength before and after polymerization. Table 3 shows the consumption percentual of CQ and TPO in the different resin-based composites. The combination of CQ and TPO increased the consumption of the photoinitiator system compared to CQ-only (p<0.001), but presented similar consumption compared to TPO-only (p=0.52).

**Table 3:** Consumption of CQ and TPO in percentage

Photoinitiator System	CQ consumption (%)*	TPO consumption (%)*
CQ	28 (3.0) B	–
TPO	–	49 (12.0) A
CQ:TPO	54 (3.0) A	51 (4.0)A

* Different letters indicate statistically significant difference in between rows.

### Degree of conversion

Table 4 shows the degree of conversion (%) of the experimental composites containing the different photoinitiator systems. There was no significant difference in the degree of conversion between the composites regardless of the photoinitiator system (p=0.81).

**Table 4:** Physical properties of the experimental composites containing the different photoinitiator systems

Photoinitiator	Degree of Conversion	Monomer Elution (µg/ml)				
System	(%)	Bis-GMA	Bis-EMA	UDMA	TEGDMA	Total
CQ	51.50 (2.3) A	0,752	1,698	0,286	0,264	3
1CQ:1TPO	51.45 (3.5) A	0,49	1,03	0	0,68	2,2
TPO	50.70 (2.9) A	8,822	3,641	0	1,337	13,8

* Different letters indicate statistically significant difference in between rows.

### Monomer elution

Table 4 also shows the monomer elution (µg/ml) of the experimental composites containing the different photoinitiator systems. The elution of the monomers was reduced when both photoinitiators were combined. TPO-based material presented the highest elution of monomers.

## Discussion

The objective of this study was to evaluate the effect of combining photoinitiators type I (mono-alkyl phosphine oxide – TPO) and II (camphorquinone – CQ) on polymerization efficiency of dental resins. The first tested hypothesis that the combination of Norrish type I and II photoinitiators would increase the consumption of the photoinitiator system was accepted. As observed in the results, the combination of camphorquinone and TPO increased the consumption of the photoinitiation system compared when the camphorquinone was used alone.

The reaction of camphorquinone with a tertiary amine result in the consumption of part of the total amount of the photoinitiator present in the material.^6^^,^^12^ As it is known, camphorquinone is a yellow-colored substance, which limits the production of certain shades, especially less yellowish shades, and bleaching shades. Also, with its consumption, a phenomenon also known as photobleaching effect occurs during the reaction. Despite the decrease in the yellow appearance of the material due to the consumption of camphorquinone, this phenomenon makes the clinical selection of color more difficult.^4^^,^^13^ TPO, on the other hand, is a whitish substance, and its combination with camphorquinone reduces the overall yellowness of the material as well as the color change throughout the curing reaction.^4^^,^^7^ Besides, this lower yellowness of the material does not only contribute to the color of the material, but better the light transmittance of the light through the material during curing.^7^^,^^9^ Thus, favoring the activation of more of the photoinitiator system, as observed in the results. Another fact that can contribute to that is the combination of camphorquinone and TPO allowed the photon absorption efficiency to increase, that is, more photons are absorbed due to the broad spectrum of the curing light used in the experiment. Thus, there is an increase in the yield of photoinitiators, especially camphorquinone.^5^^-^^6^^,^^9^

However, the second tested hypothesis that the combination of Norrish type I and II photoinitiators increases the degree of conversion of dental resin-based composites was rejected. There was no significant difference in the degree of conversion between the composites regardless of the photoinitiator system. Therefore, although the higher consumption of the photoinitiator system, the number of monomers linked to form the polymer was the same. The primary reason for this is the similar viscosity of the resin-based materials tested. Two main factors can affect the viscosity of composite materials: monomer composition and filler content. In this study, the experimental resin-based materials tested had the same components and proportions; and the degree of conversion did not change the curing process. The probability of molecular coalition at random to form longer polymer chains remained constant, reflecting in statistically similar degrees of conversion despite the different photoinitiator systems used.^10^^,^^14^^-^^15^ However, it is important to point out camphorquinone requires a co-initiator in order to react, while TPO autocleavage itself, thus differences in terms of kinetics are expected to happen. The lack of degree of conversion difference found in the current study can be associated with the fact that the maximum conversion was obtained within the imposed medium.

On the other hand, the third tested hypothesis that the combination of Norrish type I and II photoinitiators produces less elution of the monomers was accepted. The monomer elution is an important parameter to evaluate the quality of the polymeric chain formed with the presence – or not – of branches or reticulations between the polymers.^10^^-^^11^ Thus, despite the similar degree of conversion of the composites containing different photoinitiator systems, the polymeric chain formed was different. As observed in the results shown in Table 4, the polymeric chain formed by the camphorquinone alone and camphorquinone combined with TPO were more stable and less susceptible to degradation than the polymeric chain formed by the TPO system alone. The combination of camphorquinone and TPO promoted the most stable polymeric chain, with the lowest monomer elution.

The chemical structure of monomers used in the resin-based composites (Figure 1) helps explain possible polymeric chain formations and elution.^16^ The results from the nuclear magnetic resonance test are presented in a way that identifies the monomer's type through its respective characteristic functional groups.^17^ Every monomer presents a methyl methacrylate group with a double bond. By breaking this double bond, the compound will bind to a second methyl methacrylate molecule to maintain chemical stability. This process starts the chain reaction responsible for forming the polymeric chain. Dimethacrylate monomers can covalently link to four other monomers, while monomethacrylate monomers can only link to two other monomers. Thus, dimethacrylates are more likely to generate polymers with cross-linked chains, which increases the physical properties of the polymer formed.^10^

A similar degree of conversion was observed for the resin-based materials containing the different photoinitiator systems. Meanwhile, the elution of monomers when using the camphorquinone and TPO combined was 37% lower than when camphorquinone-only was used; and 52% lower than when TPO-only was used. These results indicate the polymeric network formed in the composite containing both initiators combined leads to a higher degree of crosslinking and smaller amounts of double residual bonds along the polymeric structure, explaining the lower rates of monomer elution.^18^^-^^20^

Regardless of the photoinitiator, all composites presented higher BisGMA and BisEMA elutions than the other monomers. This can be explained due to the viscosity of these monomers on their conversion. BisGMA and BisEMA have higher viscosities than UDMA and TEGDMA due to the presence of aromatic rings in the middle of the molecule (Figure 1) that significantly limits their mobility.^21^^-^^23^ The high viscosity of these monomers can interfere with their mobility and reaction with other monomers, disfavoring their conversion as the reaction occurs, and the rigidity of the polymer increases.^24^^-^^25^

It is worthwhile to mention the BisGMA is even more viscous than the BisEMA due to the presence of the -OH terminals in the BisGMA structure (Figure 1). These terminals tend to form hydrogen interactions between these monomers leading to a very high intermolecular interaction energy, thus contributing to the high viscosity of the BisGMA.^20^^,^^23^^,^^25^ This explains the higher levels of BisGMA and BisEMA in comparison to UDMA and TEGDMA. However, the higher levels of BisEMA found in the composite containing CQ and TPO combined may be due to differences in the kinetics reactive of the CQ when alone or in combination with TPO.

It is known that composites with a low level of crosslinking tend to be weaker than those with a high level of crosslinking. As a limitation of this study, the kinetics of the reaction and the crosslinking density were not evaluated. Further studies should further investigate the kinetics of the reaction and crosslinking density of composites containing CQ and TPO combined in comparison to CQ alone.

## Conclusion

Within the limitations of this *in vitro* study it was possible to conclude that the combination of the photoinitiator systems seems to be beneficial for the cure efficiency of dental resin-based composites. The combination of Norrish type I and II photoinitiators increased the consumption of the photoinitiator system; and, however it did not increase the degree of conversion of dental resin-based composites; it did reduce monomer elution.
